# Acoustic emission corrosion feature extraction and severity prediction using hybrid wavelet packet transform and linear support vector classifier

**DOI:** 10.1371/journal.pone.0261040

**Published:** 2021-12-16

**Authors:** Zazilah May, M. K. Alam, Nazrul Anuar Nayan, Noor A’in A. Rahman, Muhammad Shazwan Mahmud

**Affiliations:** 1 Electrical and Electronic Engineering Department, Universiti Teknologi PETRONAS, Seri Iskandar, Perak, Malaysia; 2 Department of Electrical, Electronic and Systems Engineering, Faculty of Engineering and Built Environment, Universiti Kebangsaan Malaysia, UKM, Bangi, Malaysia; 3 Mechanical Engineering Department, Universiti Teknologi PETRONAS, Seri Iskandar, Perak Darul Ridzuan, Malaysia; TDTU: Ton Duc Thang University, VIET NAM

## Abstract

Corrosion in carbon-steel pipelines leads to failure, which is a major cause of breakdown maintenance in the oil and gas industries. The acoustic emission (AE) signal is a reliable method for corrosion detection and classification in the modern Structural Health Monitoring (SHM) system. The efficiency of this system in detection and classification mainly depends on the suitable AE features. Therefore, many feature extraction and classification methods have been developed for corrosion detection and severity assessment. However, the extraction of appropriate AE features and classification of various levels of corrosion utilizing these extracted features are still challenging issues. To overcome these issues, this article proposes a hybrid machine learning approach that combines Wavelet Packet Transform (WPT) integrated with Fast Fourier Transform (FFT) for multiresolution feature extraction and Linear Support Vector Classifier (L-SVC) for predicting corrosion severity levels. A Laboratory-based Linear Polarization Resistance (LPR) test was performed on carbon-steel samples for AE data acquisition over a different time span. AE signals were collected at a high sampling rate with a sound well AE sensor using AEWin software. Simulation results show a linear relationship between the proposed approach-based extracted AE features and the corrosion process. For multi-class problems, three corrosion severity stages have been made based on the corrosion rate over time and AE activity. The ANOVA test results indicate the significance within and between the feature-groups where F-values (*F-value>1*) rejects the null hypothesis and P-values (*P-value<0.05*) are less than the significance level. The utilized L-SVC classifier achieves higher prediction accuracy of 99.0% than the accuracy of other benchmarked classifiers. Findings of our proposed machine learning approach confirm that it can be effectively utilized for corrosion detection and severity assessment in SHM applications.

## Introduction

Corrosion is a natural occurrence that can be defined as the decomposition of materials as a result of an environmental interaction. The resulting severe material loss leads to integrity, productivity, and financial losses [1]. Singh et al in [1] grouped corrosion’s consequences into three broad categories: safety, environmental impact, and economic impact. The structures may fail due to severe corrosion, resulting in a multitude of severe consequences. Numerous types of corrosion can occur as a result of the transportation or storage of hazardous items in corrosion-prone structures, and prior corrosion-related failures have demonstrated that they can have a significant impact on the environment and necessitate costly mitigation approaches. Corrosion has a number of economic consequences (for example, the expenses associated with repairing and maintaining corroded materials, as well as the additional costs associated with utilizing materials or protection methods to extend the lifetime of an asset).

AE is a passive Non-Destructive Testing (NDT) technology that offers a better potential in corrosion detection and monitoring. Numerous methods for detecting and assessing various types of corrosion on carbon steel materials have been published in the literature. A method based on hydrogen evolution has been devised to monitor the pitting corrosion of stainless-steel using AE in [2]. Patil et al. [3] designed a technique for evaluating accelerated corrosion tests based on AE. In addition, Prateepasen et al. [4] utilized acoustic emission to detect pitting corrosion. Accelerated corrosion testing was performed on SS-304 specimens that had been ground with silicon carbide paper, rinsed with distilled water, and dried in the air in the proposed technique. Droubi et al. [5] suggested a method for predicting corrosion using AE. Time domain analysis were performed on AE signals and discovered a link between AE Energy and corrosion. Saenkhum et al. [6] classified corrosion using acoustic emission and an Artificial Neural Network (ANN). Four characteristics experiment-derived AE energy, amplitude, rising time, and count were employed as inputs to a neural network. The testing phase of the neural network has a very low rate of misclassification and an excellent capacity for generalization, with training accuracy of 96.41% and testing accuracy of 94.35%. De Masi et al. [7] used a Fitting Neural Network (FNN)-based regression approach to predict the rate of corrosion, metal loss, defect area, and defect count in subsea pipelines. Liao et al. [8] employed hybrid machine learning algorithms such as Genetic Algorithm (GA) optimized Back-Propagation Neural Network (BP-NN) and Particle Swarm Optimization (PSO) optimized BP-NN to predict the numerical corrosion rate of gas pipelines during the internal corrosion assessment process. For corrosion rate prediction, the network was trained with seven input neurons, fourteen hidden layer neurons, and a single output neuron. On the basis of its lowest absolute error, GA optimized BP-NN demonstrated the best corrosion prediction rate compared to other techniques. In 2009, Piotrkowski et al. [9] used wavelet analysis (WA) and bi-spectrum analysis (BA) on AE signals to identify and evaluate corrosion damage in galvanized steel. Griffin et al. [10] used both the Short-Time Fourier Transform (STFT) and the Wavelet-Packet Transform (WPT) on AE signals extracted during burn and chatter anomalies. In [11], Zhao et al. used wavelet packet analysis (WPA) and support vector machines (SVM) to classify AE signals in composite laminates. In [12], Van Dijck and Van Hulle identified corrosion absence, uniform corrosion, pitting, and stress corrosion cracking using a hybrid filter-wrapper genetic algorithm and a naive Bayes classifier. Yu and Zhou suggested a method for classifying AE signals resulting from oil storage tank damage in [13], combining SVM and an optimized grid search algorithm, whereas Li et al. in [14] used K-means clustering to classify AE signals generated by 304 stainless steel during the stress corrosion process. Hybrid machine learning approaches are utilized that combines corrosion detection using AE signals from accelerated corrosion testing with a machine learning algorithm to provide an accurate prediction of corrosion severity levels in [15, 16]. The AE technique is introduced in [17] to monitor the corrosion process and cracking behaviour in large-scale reinforced concrete (RC) pile specimens in the marine environment. Moreover, AE-based SHM technique in [18] is applied to improve the level of safety in aircraft. However, some of the issues are being faced or may be faced while bringing in corrosion into the SHM framework for aircraft are highlighted. These studies, with the exception of few of them, are based on supervised learning algorithms. Thus, structure-borne AE assessed using supervised learning-based algorithms are reliable methods for corrosion detection and evaluation. Nevertheless, machine learning techniques for feature extraction and classification of corroded AE signals are still in their infancy.

There are several categories of AE features (statistical, time-domain and frequency-domain) extracted by different methods in order to utilize them for corrosion detection and severity level prediction. However, the existing methods are not addressed to extract features from the multiresolution signal that may reduce the features variability and resulting degrade the diagnosis ability in the corrosion detection and assessment system. This article aims to develop a new hybrid machine learning approach including WPT integrated with FFT to process AE signals and extract three categories of AE features, and the supervised learning model L-SVC to predict the severity levels of corrosion. Initially, the AE signal is acquired from the LPR test experiment using the AEWin system and categorized the severity of corrosion into three different classes based on corrosion rate over various time spans and corrosion activity. Then, the extracted signals are pre-processed and decomposed signal by WPT to generate a large number of time-frequency multiresolution wavelet packets based on a defined decomposition level to extract statistical and time-domain features for three different classes. Afterwards, the decomposed wavelet packets in each level are transformed into the frequency domain by FFT to extract the frequency-domain features for all classes. Then, the L-SVC is built with the optimal parameters and trained utilizing our extracted feature sets for three classes. Finally, the trained model is tasted with our test feature sets to evaluate the prediction performance. Various performance indicators are used to measure the performance of our model as well as well-known benchmarked models.

## Materials and experiments

This section explains the various features and characteristics of the given specimens. The details of the experimental tests for mechanical and AE signal acquisition are discussed. The potential applied was in the range ±0.25 V respect to the reference electrode.

### Linear Polarization Resistance (LPR) test

**Fig 1:**
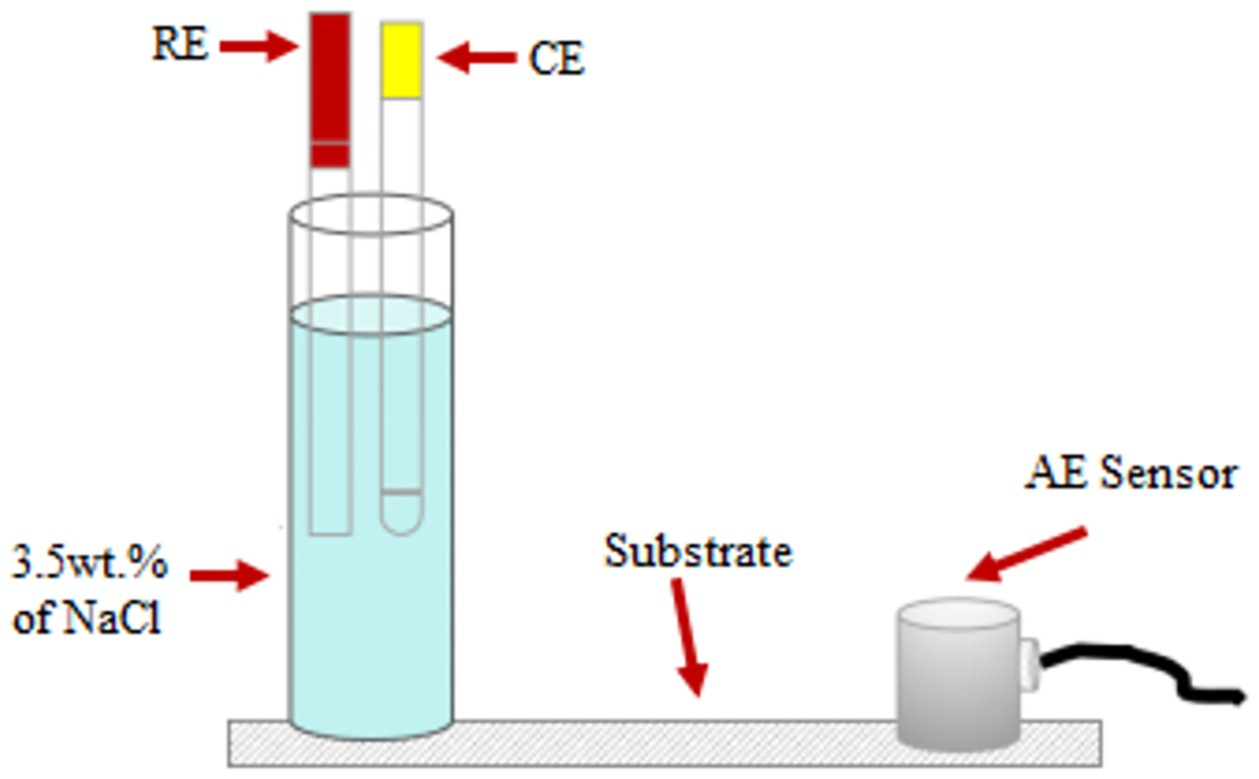
Corrosion test set-up, connected to AE sensor and potentiostat for acquisition.

**Fig 2:**
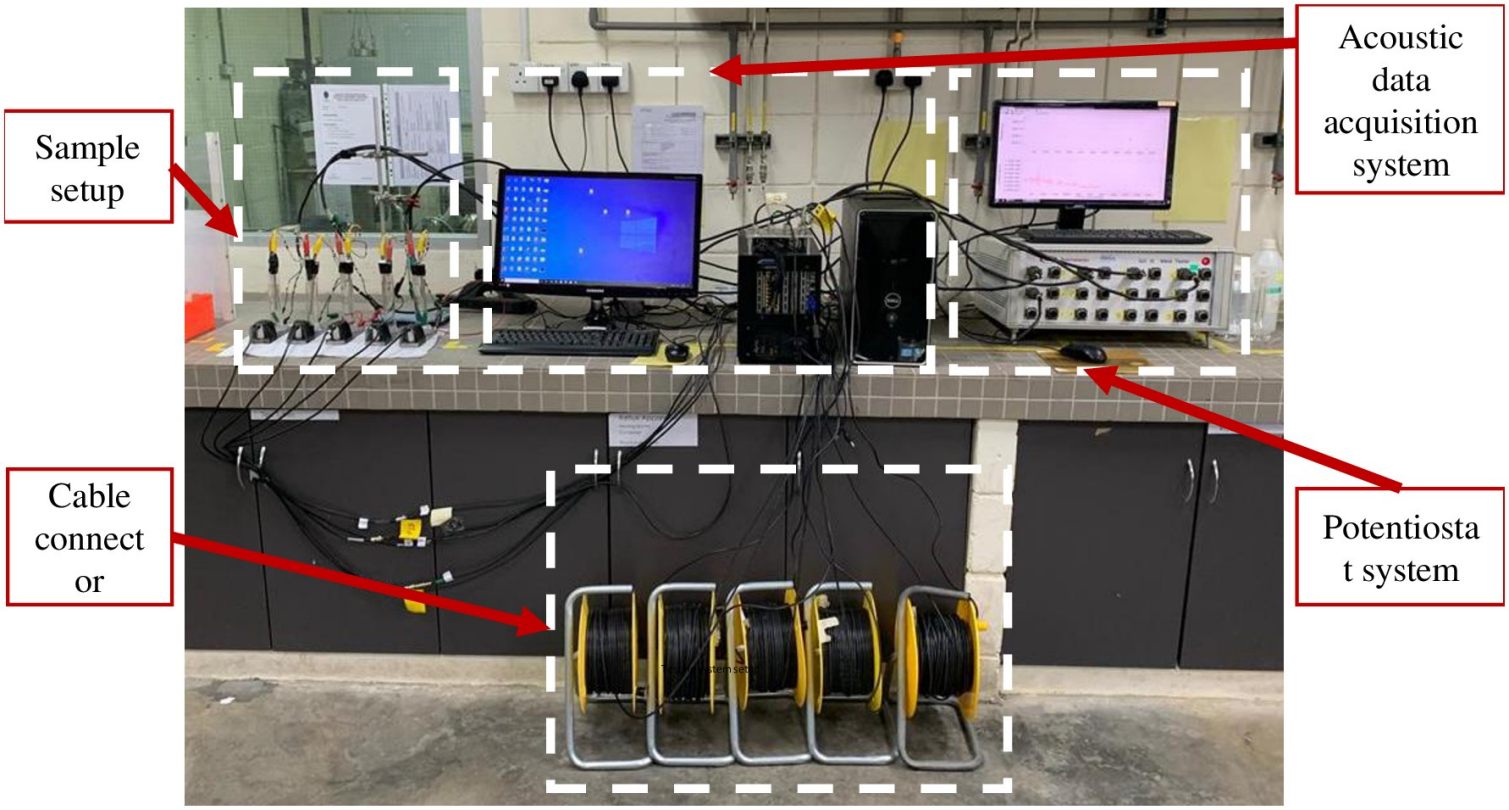
Carbon-steel LPR test experimental system.

LPR is an electrochemical test commonly used in material corrosion studies to gain corrosion rate data at a potential range between ±0.01 V respect to the reference electrode. The measurement was carried out by using an ACM Gill potentiostat. In the three-electrode system, a carbon steel substrate with a dimension of 150 x 50 x 2 mm is assigned as the working electrode (WE), a stainless steel rod as the counter electrode (CE), while Ag/AgCl is assigned as the reference electrode (RE) in the system. The electrolyte used in this work is 3.5 wt.% of sodium chloride. A portion of the substrate is exposed to the electrolyte with a surface area of approximately 2.86 *cm*^2^. The exposed area is isolated by using an acrylic rod. Details of the connection of this sample setup are shown in Fig 1. The acoustic emission sensor was attached to the other end of the substrate. Moreover, Fig 2 illustrates the complete diagram of the whole system during data acquisition. During the polarization, the DAQ system of potentiostat recorded the corrosion data, including corrosion rate, for up to 72 hours.

### Acoustic emission signal acquisition

**Fig 3:**
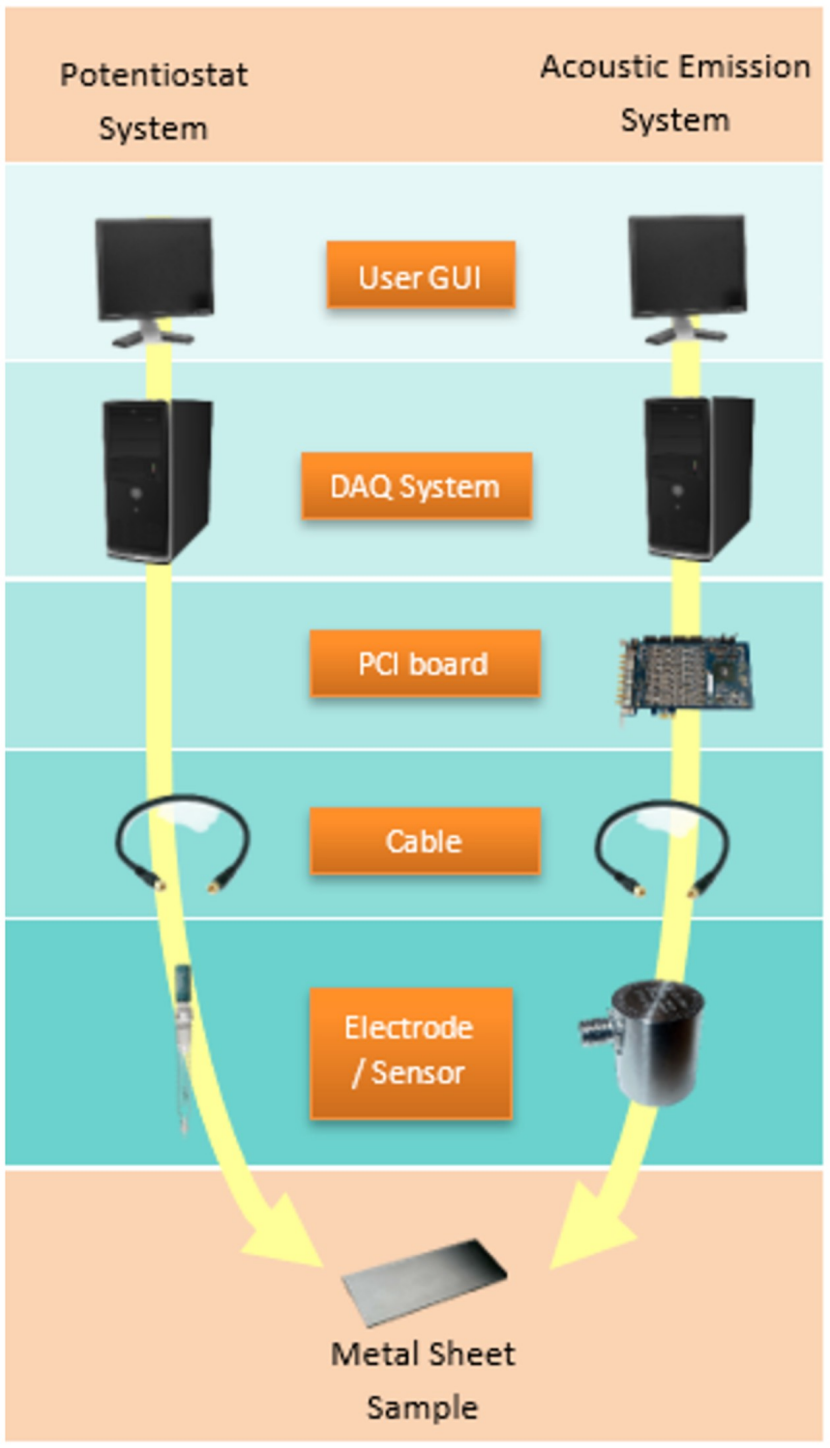
Schematic flow diagram of LPR test during AE data acquisition.

AE signals were acquired continually throughout the test. Fig 3 describes the schematic flow of AE data acquisition using the LPR test. Signals were monitored using four data collecting channels and an environmental noise test. Magnetic clamps are used to secure the sensors to the specimen. Between the sensors and the specimen, a coupling agent is used to significantly increase the amount of acoustic energy transmitted from the specimen to the sensor. Physical Acoustics Corporation (USA) supplied the entire system, including the sensors. Prior to data collection, the Pencil lead break process was used to calibrate and guarantee that all sensors received the maximum amplitude from the lead break. The acquisition configuration used the values for peak definition time (PDT), hit definition time (HDT), and hit lockout time (HLT), as well as a threshold value and sample rate, as stated in Table 1. The sensors were placed on the pipe specimen as illustrated in Fig 1.

**Table 1:** AE parameters.

Parameter	Value
Hit definition time (HDT)	2000 μs
Peak definition time (PDT)	1000 μs
Hit lockout value (HLT)	500 μs
Threshold value	40 dB
Sample rate	1 μs per sample

R1.5I-AST sensors are employed to acquire the AE signal and provide components for high-sensitivity data acquisition and recording in this work. Table 2 summarizes the sensor specification. Before and during the experiment, data on the normal (ground-truth) and abnormal (cathodic charging) AE signals were collected, respectively. Charging and data collection take around 146 seconds in total, with a sampling rate of 1μs per sample. Thus, each AE hit generated a 1024-line data set. AEwin software was used to acquire and record the data, which included all waveform features. The wavelet packet transform, empirical mode decommissioning, and other techniques were used to evaluate and process the AE features.

**Table 2:** Specifications of R1.5I-AST sensor.

Parameter	Value
Peak sensitivity, ref (V/(m/s))	124 dB
Operating frequency Range	5-20 kHz
Resonant Frequency, ref (V/(m/s))	14 kHz

## Methodology of the AE features extraction and classification approach

**Fig 4:**
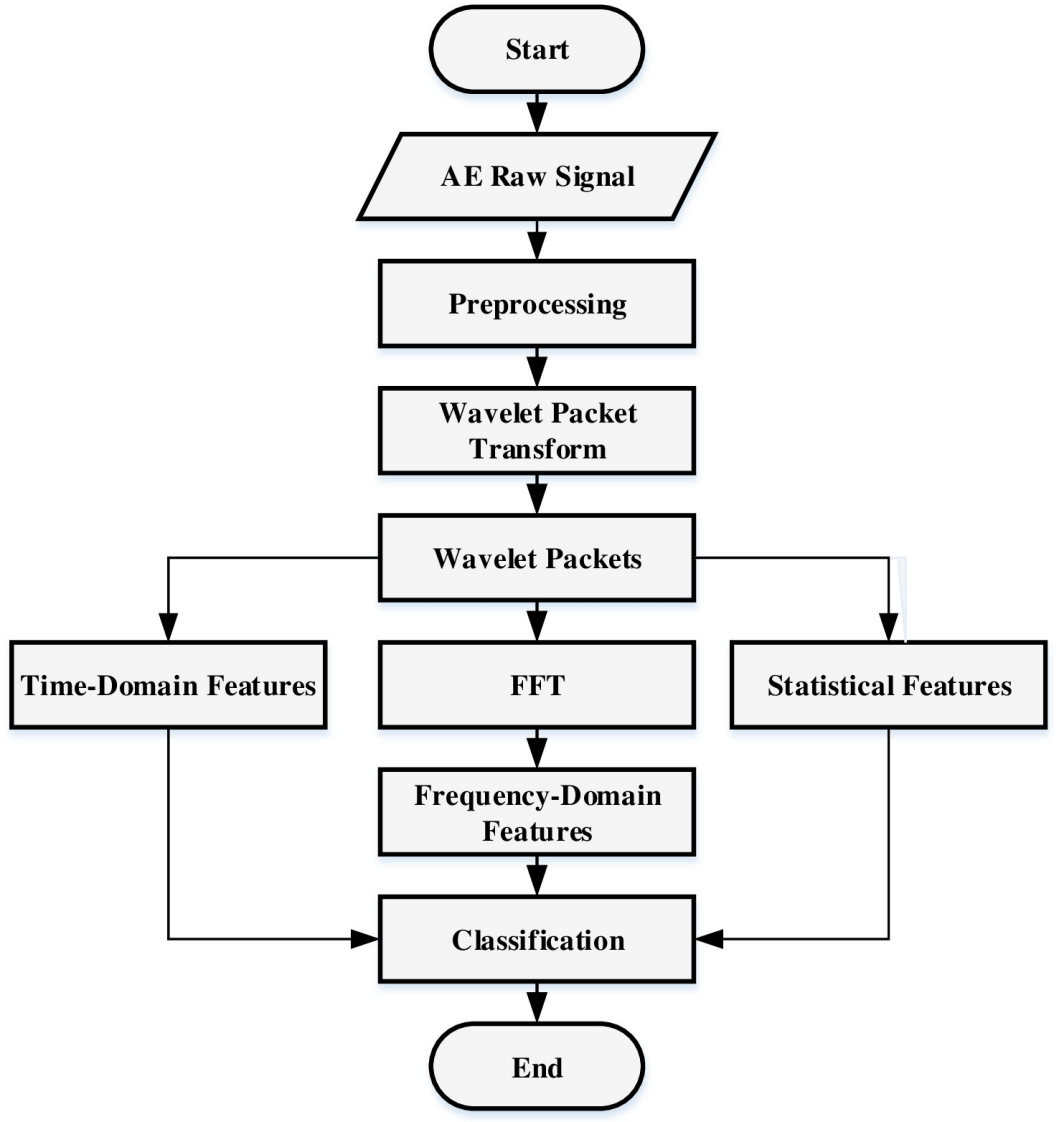
A flowchart of the proposed AE features extraction and classification approach.

The acquired AE signals are used as input to the proposed signal feature extraction and classification approach for carbon-steel corrosion assessments. The overall flowchart of the proposed approach is presented in Fig 4.

### AE signal preprocessing

**Fig 5:**
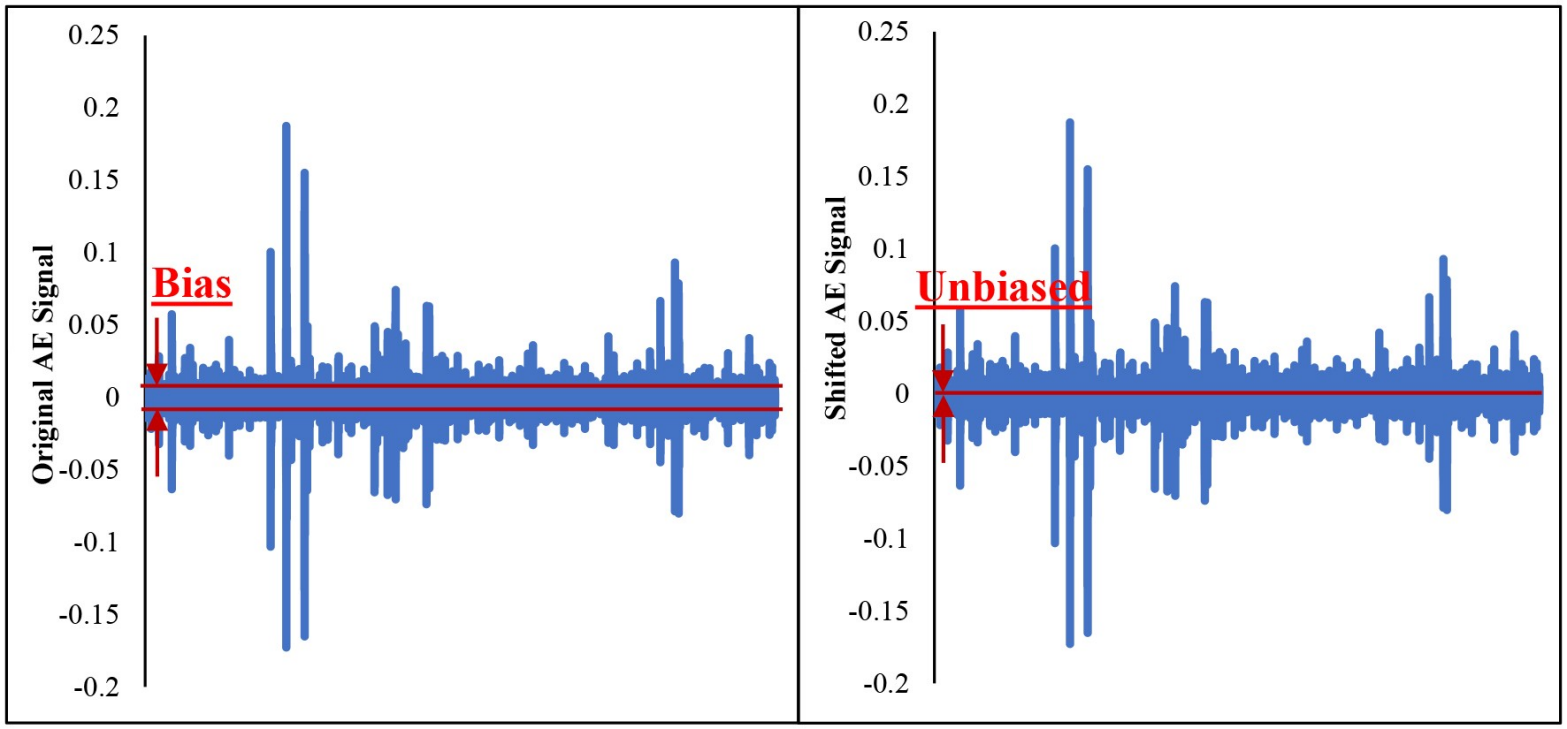
Bias original and unbiased shifted AE signals.

The AE raw signal is usually recorded as an individual file containing a single waveform at a specific time. The recorded files over time are appended in a matrix form in the preprocessing stage in order to be processed further. According to the study in [19], typically, AE raw signals oscillate around zero, resulting in a zero mean. However, the obtained AE raw data exhibit a bias consisting of an offset likely caused by electrical noise generated by the AE sensor system. It is needed to remove bias during the preprocessing phase by shifting the AE raw signals to achieve a zero mean signal, as depicted in Fig 5. For each AE raw signal (Fig 5), the average value of the signal was determined and subtracted from the original signal to obtain the characteristic AE raw signal oscillating around zero in the unbiased shifted AE signal. Finally, the shifted original signals are cleaned based on our previously published denoising method in [20].

### Features of acoustic emission signal

**Fig 6:**
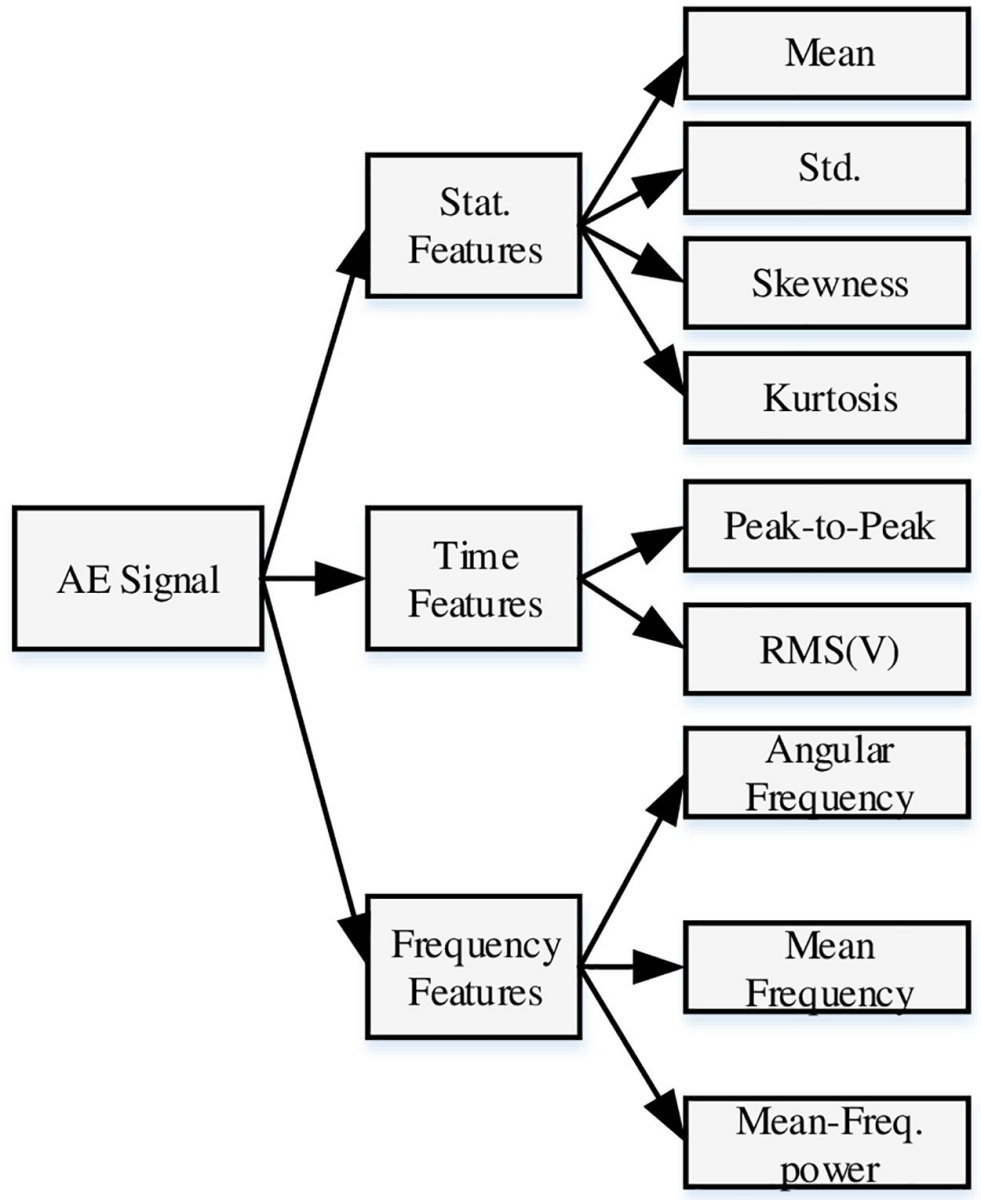
Different types of AE features.

The AE features are mostly utilized to examine the structures, assess the materials and monitor the manufacturing processes. The AE features can mainly be divided into three categories, as detailed in Fig 6.

The signal mean, standard deviation, skewness, and kurtosis were selected as statistical analysis features and peak-to-peak amplitude as well as signal rms energy were chosen as time-domain features to be extracted from the pre-processed AE signals. Moreover, the angular frequency, mean frequency, and mean frequency power were considered as frequency-domain features to be extracted from the FTT-transformed AE signals. These features make up the elements of analysis pattern vectors to be in-putted into pattern recognition paradigms for decision making on materials’ health acceptability.

### Wavelet packet decomposition

Wavelet packet decomposition subdivides the frequency band into many levels and further subdivides the high-frequency portion of the band that is not subdivided by wavelet analysis. Wavelet packet decomposition selects the appropriate frequency band adaptively to match the signal’s spectrum properties, which enhances time frequency resolution. According to the random time frequency resolution, the wavelet packet decomposes the signal into the corresponding frequency band components. The wavelet packet approximation formulation is obtained by performing a multi-resolution analysis on the square integrable real space as following.

$$\begin{array}{c} L^{2}\left(R\right)=⊕W_{-1}⊕W_{0}⊕W_{1}⊕={⊕}_{f\in z}W_{j},\forall \in Z \end{array}$$ (1)

where the space of the wavelet function is *W*_*j*_, the scale factor is *j*, ⊕ is the orthogonal sum of the two subspaces. Eq 1 means that the space of the real number, *L*^2^(*R*), is the orthogonal sum of the wavelet subspace *W*_*j*_ where *j* ∈ *z* according to different scale factors. Wavelet packet analysis can improve the frequency resolution by subdividing the frequency band into binary form.

**Fig 7:**
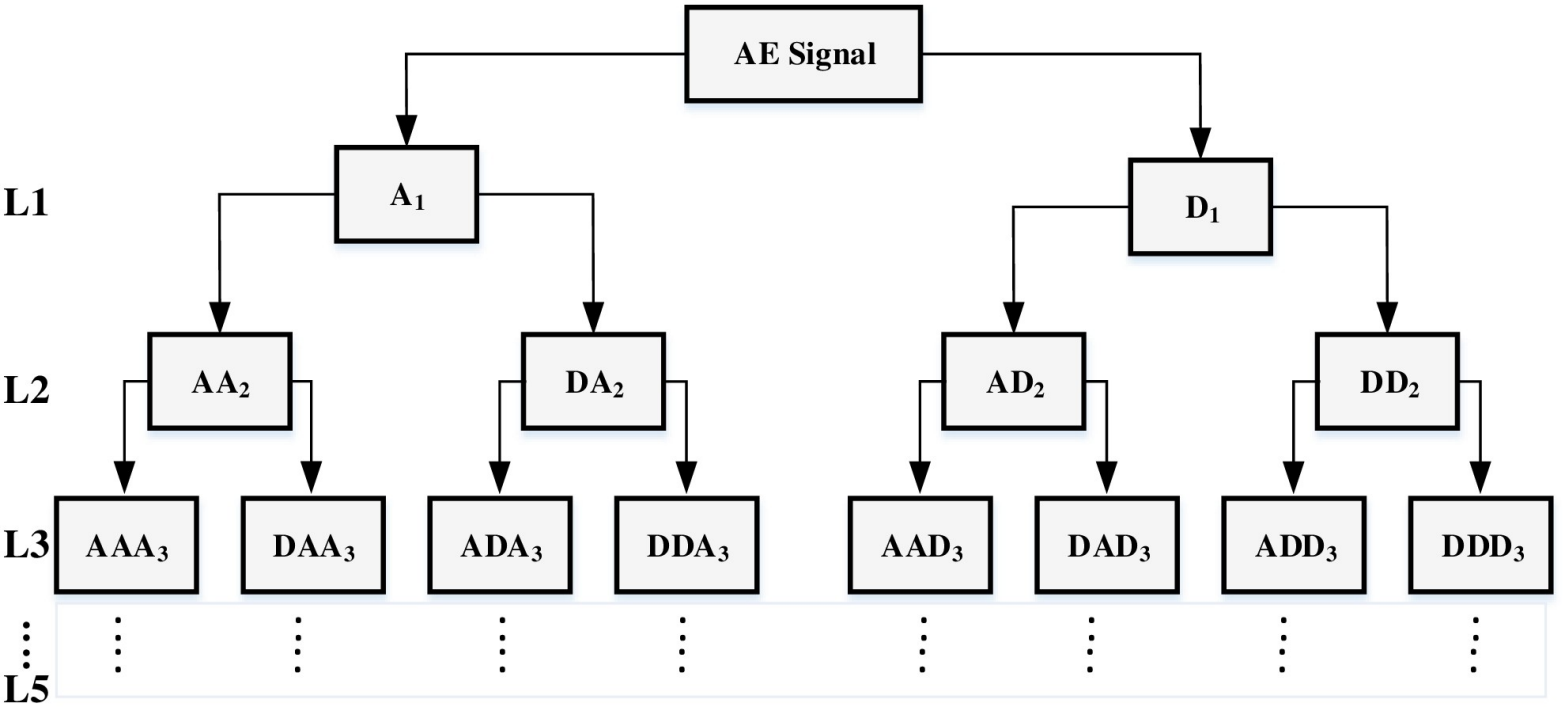
Five-layers wavelet packet decomposition tree.

A signal’s WPT generates packets of coefficients calculated by scaling and shifting a specified mother wavelet, which is a prototype function. As a result, at the WPT’s first level, the original signal S is divided into two frequency band packets referred to as approximation, *A*_1_, and detail, *D*_1_. At the 2nd level, each approximation and detail packet are again split into further approximations, *AA*_2_ and *AD*_2_, and details, *DA*_2_ and *DD*_2_, and the process is repeated in the next levels, generating other decomposition packets as presented in Fig 7. The mother wavelet employed for WPT of the pre-processed AE signals is a Coefficient 5, denoted by “coif5”. The decomposition was performed up to the 5th level, yielding 62 packets. For each packet, 4 statistical, 2 time-domain and 3 frequency-domain features were calculated.

### Linear Support Vector Classifier (L-SVC)

**Fig 8:**
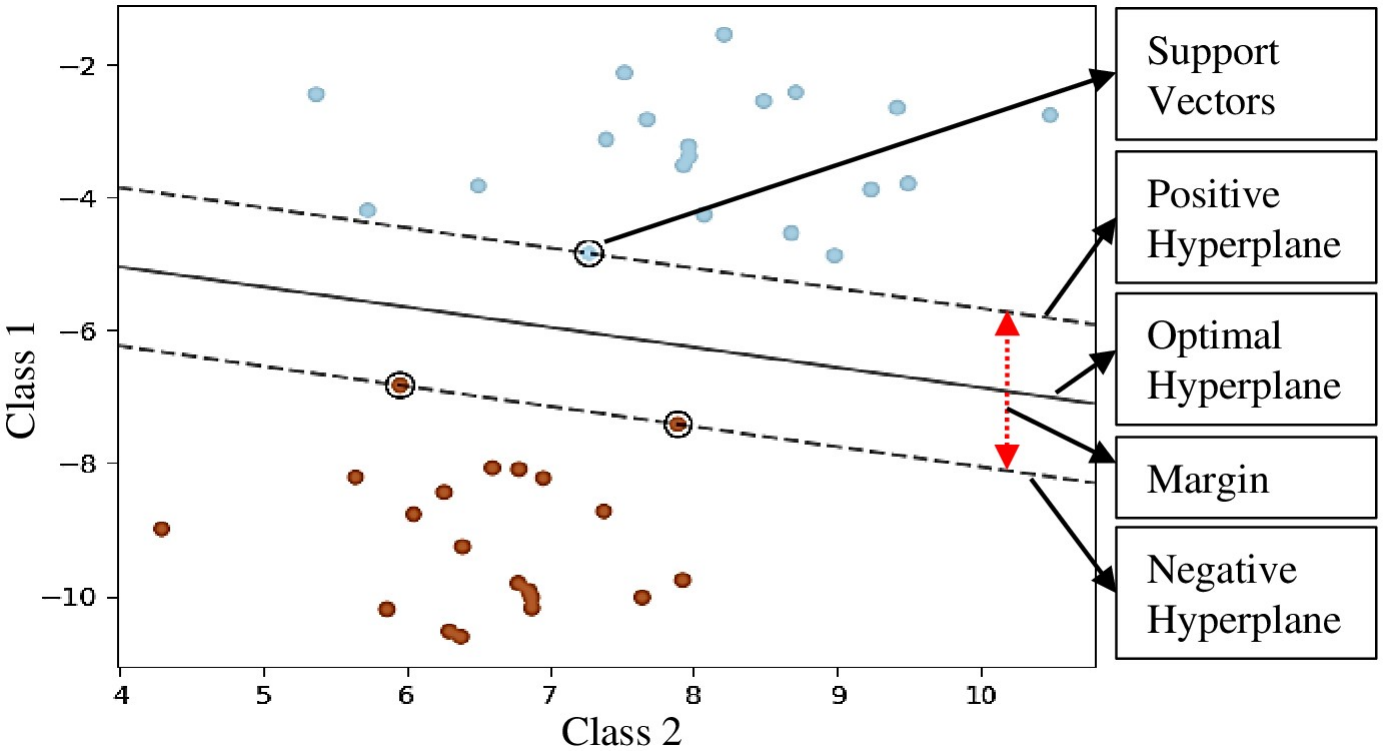
Clustering processes of Support Vector Classifier (SVC).

Support Vector Classifier (SVC) is a supervised machine learning technique which constructs a hyperplane or set of hyperplanes in a high or infinite dimensional space. It can be utilized for classification, regression, clustering, and detection tasks. Generally, the optimum clustering is achieved while the hyperplane has the maximum distance to the nearest training data-points of any class. The distance between the nearest training data points of a class and the data points of another class is also known as the functional margin. In general, a large margin introduces a small generalization error of the classifier. Fig 8 presents the decision function of the linear clustering problem for two classes.

Considering a binary classification problem with a training dataset, where given training set $X\in R^{n}$ in a set of classes *Y* = {Φ_1_, Φ_2_, ⋯, Φ_*k*_} where *k* ≥ 2 is an integer. Binary and multi-class classification depend on the *k* value, *k* = 2 is considered as binary and *k* > 2 is referred as multi-class classification. SVC is one of the most promising algorithm which can be used for both classes classification whereas the Support Vector Machine (SVM) works well for binary class classification [21]. Based on independently and identically distribution, the training set is as in Eq 2.

$$\begin{array}{c} T=\{(x_{1},y_{1}),(x_{2},y_{2}),\cdots (x_{n},y_{n})\}\in {\left(X\times Y\right)}^{n} \end{array}$$ (2)

where *x*_*i*_ and *y*_*i*_ are the input and output training vectors of a same class and *i* = 1, 2, ⋯, *n*.

The standard SVC framework in linear programming formulation is explained briefly here before formulating the L-SVC framework [21]. Based on Eq 2, we solve the binary classification problem where input support vector *x*_*i*_ ∈ *X* and output support vector *y*_*i*_ ∈ *Y* = {−1, 1}. The main objective of SVC framework is to find a hyperplane (*w*⋅*x*) + *b* = 0, where the largest margin $w\in R^{n}$ and the independent term b∈R. The computed hyperplane is utilized to separate two classes with the largest margin in binary classification. The correct prediction is given for most observations by sign (*w*^*T*^ ⋅ Φ(*x*) + *b*). Standard SVC solves the primal problem as follows:

$$\begin{array}{c} \underset{w,b,\xi }{\text{min}}\;\frac{1}{2}{\left\|w\right\|}^{2}+C\sum_{i=1}^{n}\xi_{i}\;, \end{array}$$ (3)

$$\begin{array}{c} \text{s.t.}\;{\text{y}}_{i}(w^{T}\cdot \Phi (x_{i})+b)\geq 1-\xi_{i}\;, \end{array}$$ (4)

$$\begin{array}{c} \xi_{i}\geq 0\;,\;\text{i = 1,}\cdots ,\text{n} \end{array}$$ (5)

In Eq 3, we maximize the margin *w* by minimizing (‖*w*‖^2^ = *w*^*T*^ *w*). The misclassification is considered within the margin boundary while incurring a penalty where the margin distance and penalty term are defined as *ξ* and *C* correspondingly. Based on Eq 4, The perfect prediction is considered when the value *y*_*i*_ (*w*^*T*^ ⋅ Φ(*x*_*i*_) + *b*) is greater than or equal to 1.

Now, we formulate binary class classification problem for L-SVC which is utilized in our work. Based on the training set in Eq 2, we define the input vector *x*_*i*_ ∈ *X* and *y*_*i*_ ∈ *Y* = {1, 2} as well as find a matrix *W* = (*w*_1_, *w*_2_) which satisfied the Eqs 6 and 7.

$$\begin{array}{c} (w_{1}\cdot \Phi (x_{i}))\geq (w_{2}\cdot \Phi (x_{i}))\;{\text{y}}_{i}=1; \end{array}$$ (6)

$$\begin{array}{c} (w_{2}\cdot \Phi (x_{i}))\geq (w_{1}\cdot \Phi (x_{i}))\;{\text{y}}_{i}=2; \end{array}$$ (7)

where *y* = *f*(*x*) = arg max_*r*=1,2_ (*w*_*r*_ ⋅ Φ(*x*)) and its optimization problem is formulated as in Eqs 8, 9, 10 and 11.

$$\begin{array}{c} \underset{w,\xi }{\text{min}}\;\frac{1}{2}({\left\|w_{1}\right\|}^{2}+\|w_{2}\|)+C\sum_{i=1}^{n}\xi_{i} \end{array}$$ (8)

$$\begin{array}{c} \text{s.t.}\;(w_{1}\cdot \Phi (x_{i})-w_{2}\cdot \Phi (x_{i}))\geq 1-\xi_{i}\;,\;y_{i}=1 \end{array}$$ (9)

$$\begin{array}{c} (w_{2}\cdot \Phi (x_{i})-w_{1}\cdot \Phi (x_{i}))\geq 1-\xi_{i}\;,\;y_{i}=2 \end{array}$$ (10)

$$\begin{array}{c} \xi_{i}\geq 0\;,\;\text{i = 1,}\cdots ,\text{n} \end{array}$$ (11)

Accordingly, we can formulate our L-SVC framework with kernel.

$$\begin{array}{c} \underset{\alpha^{1},\alpha^{2},\xi }{\text{min}}\;\;\text{-}\sum_{i=1}^{n}(\alpha_{i}^{1}+\alpha_{i}^{2})+C\sum_{i=1}^{n}\xi_{i} \end{array}$$ (12)

$$\begin{array}{c} \text{s.t.}\;\sum_{i=1}^{n}\alpha_{i}^{1}K(x_{i},x_{j})-\sum_{i=1}^{n}\alpha_{i}^{2}K(x_{i},x_{j})\geq 1-\xi_{i}\;,\;y_{i}=1 \end{array}$$ (13)

$$\begin{array}{c} \sum_{i=1}^{n}\alpha_{i}^{2}K(x_{i},x_{j})-\sum_{i=1}^{n}\alpha_{i}^{1}K(x_{i},x_{j})\geq 1-\xi_{i}\;,\;y_{i}=2 \end{array}$$ (14)

$$\begin{array}{c} \alpha_{i}^{r},\;\xi_{i}\geq 0\;,\;i=1,\cdots ,n\;,\;\text{r = 1,2} \end{array}$$ (15)

where the *n* by *n* positive semi-definite matrix is defined as *Q*_*ij*_ ≡ *y*_*i*_ *y*_*j*_ *K* (*x*_*i*_, *x*_*j*_) and *K* (*x*_*i*_, *x*_*j*_) = Φ(*x*_*i*_)^*T*^Φ(*x*_*j*_) is considered as the kernel. The *α*_*i*_ is defined as dual coefficient and they are upper-bounded by *C*.

Following the above binary classification and optimization equations, we can extend them to solve multi-class classification and optimization problems. When the optimization problem is resolved, the output of the decision function for a given observation *x* is derived as in Eq 16.

$$\begin{array}{c} \sum_{i\in sv}y_{i}\alpha_{i}K(x_{i},x)+b \end{array}$$ (16)

## Datasets and L-SVC model specifications

The description of the AE datasets, simulation environment, and utilized parameters in the L-SVC model are presented in this section. Moreover, the performance of the proposed work is analyzed based on several performance metrics.

### Acoustic emission datasets

**Fig 9:**
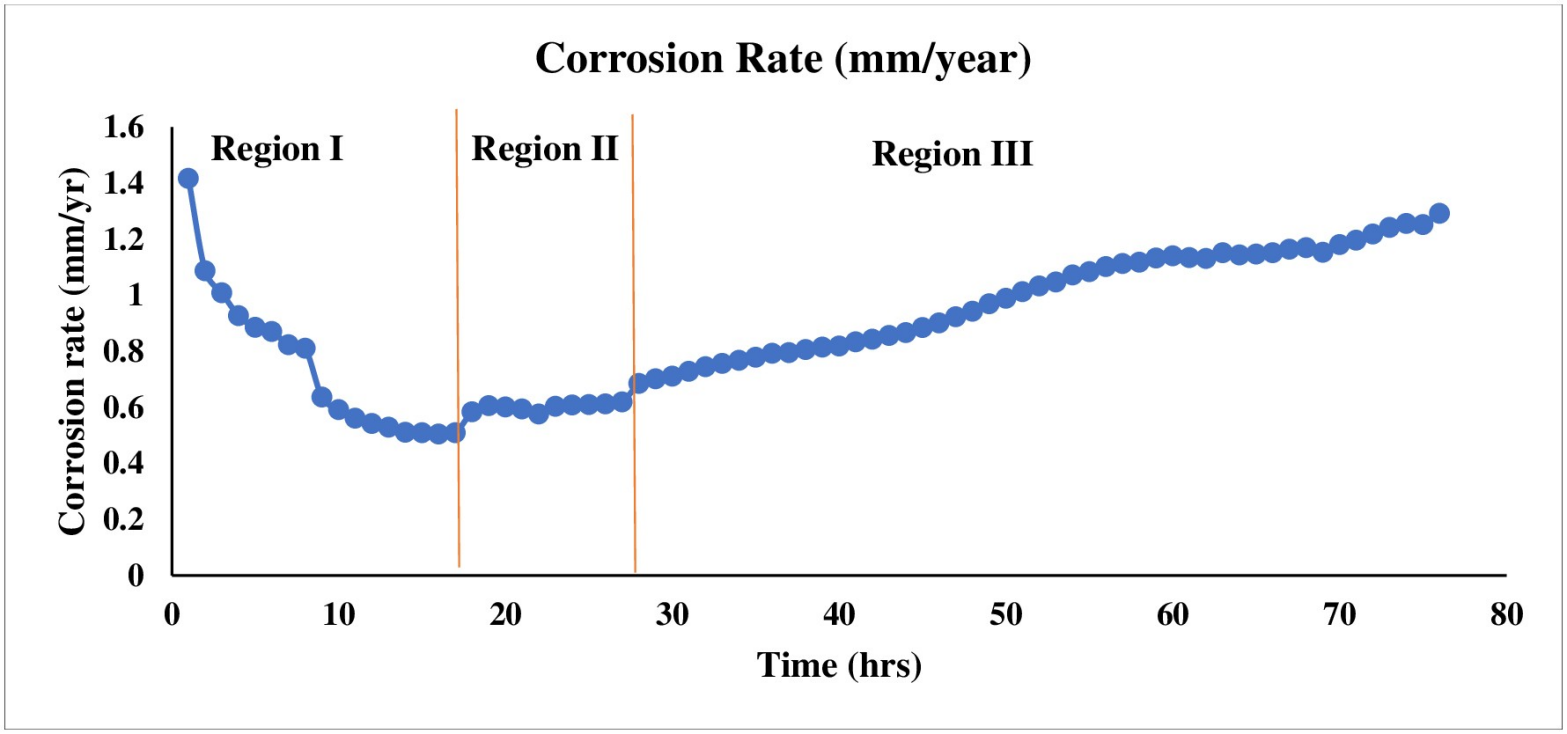
Categorization of severity levels of uniform corrosion.

**Fig 10:**
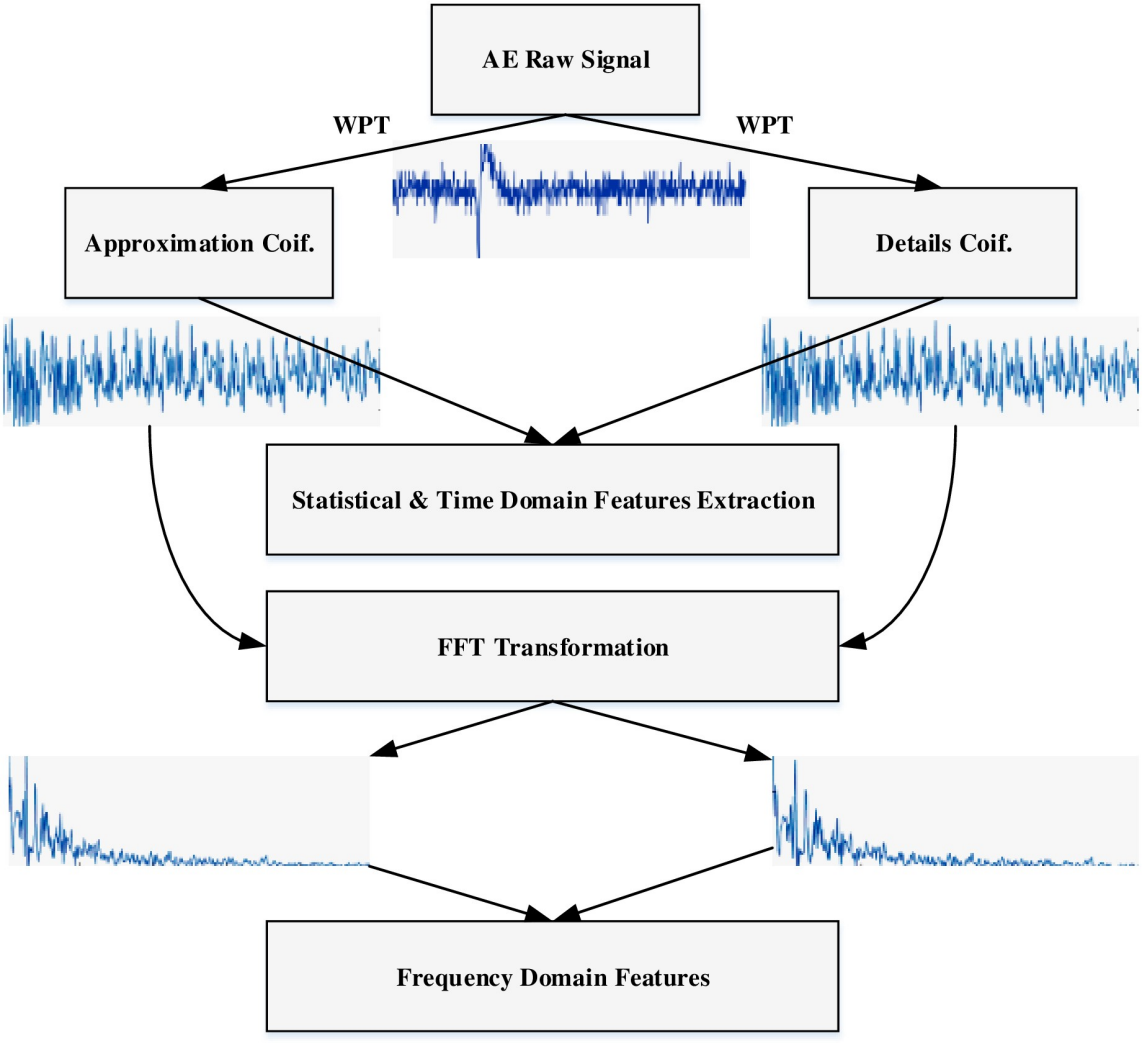
Feature extraction process of AE datasets for machine learning model.

The AE corrosion datasets were acquired from the carbon-steel LPR test experimental system. A single AE sensor was placed on a carbon-steel substrate for data collection. The AE data was recorded every microsecond as waveforms for a duration of approximately 72 hours. Each waveform duration was 2 milliseconds and it represented a single measurement (AE signal amplitude in voltage). The generation of AE waveforms mainly depends on corrosion activity and predefined threshold. The threshold value was set at 25 dB in the LPR test experiment. There is an inversely proportional relationship between corrosion activity and waveform generation. Thus, the number of recorded waveforms may vary every hour. The total duration of recorded AE data was categorized into three different levels of corrosion based on the corrosion rate over a different time span and corrosion activity presented in Fig 9. “Region I” is defined as an initial level of corrosion whose duration is between 1 to 17 hours. “Region II” and “Region III” are considered average and severe levels of corrosion. Their duration is between 18 to 29 and 30 to 72 hours respectively. In “Region I”, 435 waveforms were generated. Each waveform contains 2048 samples, for a total of 890880 data samples. In “Region II”, 36 waveforms were recorded and a total of 73728 samples, whereas “Region III” contained 375 waveforms and a total of 768000 data samples. The mass loss measured by corrosion rate for region I are decreasing from 1.4mm/yr down to 0.3mm/yr. The descending trend is a natural process by the metal during building up an oxide layer at the beginning of immersion process. The mass loss at Region II become stable compared to Region I, which are between 0.6 to 0.7 mm/yr. This confirms the existence of temporary oxide layer form on the specimen after 17 hours. Meanwhile, the increasing of corrosion rate observes in Region III from 0.7 to 1.3 mm/yr indicates that the layer was ruptured due to the aggregative charge/ion transfer between electrolyte and the specimen. The raw AE data collected from all regions was denoised based on the ND-SWT method and used to obtain feature datasets of three regions for our machine learning model. The five-level WPT decomposition method was utilized to decompose AE cleaned signals for all regions individually. Each region AE signal was decomposed into two different types of packets called “approximation coefficient” and “detail coefficient” in five-level generated total of 62 packets. Three statistical features and two time-domain features were extracted from these 62 packets as shown in Fig 10. In order to compute three frequency-domain features, each packet generated from WPT decomposition was transformed based on the Fast Fourier Transform (FFT) as presented in Fig 10 as well. The extracted features from three domains were formulated as a matrix shaped (62 × 9) where a number of rows refers to samples and a number of columns presents AE features. Table 3 represents an example of a features set for a single region which is utilized as an input dataset for our L-SVC model.

**Table 3:** An example of AE features set of three domains for single region.

Features No.	Time Domain			Statistic Domain			Frequency Domain		
	P2P (V)	RMS (V)	Mean	Std.	Skewness	Kurtosis	Mean Freq.(kHz)	Mean Freq. P. (dB	Angular Freq.(kHz)
1	0.11	0.4E-3	-0.4E-3	0.2E-2	-1.07	40.	80.76	-116.9	702.3
2	0.09	1.3E-7	0.1E-6	0.001	-0.02	240	80.76	-116.9	702.6
3	0.09	0.7E-3	-0.7E-3	2.8E-3	-0.90	18.4	59.86	-111.9	455.1
:	:	:	:	:	:	:	:	:	:
62	.04	2.9E-7	0.2E-6	1.1E-3	2.6E-1	31.9	6.27	-100.9	64.90

### L-SVC model simulation specifications

The L-SVC is a supervised machine learning model which is utilized as a multi-classes classification framework for different levels of corrosion prediction in our work. The L-SVC model has been implemented in the Python environment for training and testing performance evaluation based on our input labelled dataset. There are 9 feature variables, each containing 62 samples, and 1 target variable which contains three different classes labelled as “Region I:1”, “Region II:2” and “Region III:3” correspondingly. The input dataset is split into two parts, 70% for training and 30% for testing. A summary of the L-SVC model specifications utilized for simulation is listed in Table 4.

**Table 4:** A summary of L-SVC model specifications utilized for simulation.

Parameter	Value
Input dataset	186 x 9
Model	LinearSVC
Library	SVM
Penalty	l2
Loss	squared_hinge
Dual	False
Tolerance	1*e*^−4^
Regularization	1.0
Multi_class	“ovr”
Max_iter	3000
Cross-validation	10-fold
Framework	Scikit-learn
Environment	Python 3.8 (Anaconda)

## Results and discussion

The proposed feature extraction and classification approach has been implemented using the scikit-learn framework in the Python anaconda environment and simulated using the LPR test experimental corrosion dataset. The utilized corrosion dataset consists of three different levels of corrosion and the combination of statistical, time-domain and frequency-domain features for all classes are extracted based on our proposed feature extraction method. In this section, we investigate the extracted feature correlation and the importance of the features in predicting target variables based on the sum of input feature coefficients in order to make a prediction. Moreover, we investigated the performance of the L-SVC multi-class classification model and compared it with the performance of other well-known prediction models named Support Vector Machine (SVM), Decision Tree (DT), Logistic Regression (LR) and Random Forest (RF). The performance evaluation is carried out based on various performance metrics listed as follows:

### Precision

The precision is computed by the ratio of predicted True Positive (TP) value of an individual class and sum of the TP as well as False Positive (FP) of that particular class. The precision can be defined as in Eq 17.

$$\begin{array}{c} Precision=\frac{\left(TP\right)}{\left(TP+FP\right)} \end{array}$$ (17)

### Recall

The recall is calculated by the ratio of TP prediction of one class and the sum of the TP predictions of that particular class as well as False Negative (FN) of another class. The recall can be formulated as follows:

$$\begin{array}{c} Recall=\frac{\left(TP\right)}{\left(TP+FN\right)} \end{array}$$ (18)

### F1-score

The F1-score is computed between 0 and 1 which is the harmonic mean of the precision and recall. The F1-score can be defined as in Eq 19.

$$\begin{array}{c} F1-Score=\frac{2\times (precision\times Recall)}{\left(Precision+Recall\right)} \end{array}$$ (19)

### Accuracy

The accuracy is counted by the ratio of the sum of TP and TN predictions of all classes and the sum of TP, FP, TN as well as FN of all classes. The accuracy can be formulated as in Eq 20.

$$\begin{array}{c} Accuracy=\frac{\left(TP+TN\right)}{\left(TP+FP+FN+TN\right)} \end{array}$$ (20)

### Mean Absolute Error (MAE)

The MAE is calculated by finding the average of absolute difference between the actual values and predicted values. It can be formulated as in Eq 21.

$$\begin{array}{c} MAE=\frac{1}{n}\sum_{i=1}^{n}(|\text{Actual}\;\text{values}\;\text{-}\;\text{Predicted}\;\text{values}|) \end{array}$$ (21)

where *n* is the total number of actual or predicted values and *i* ∈ {1, *n*}.

In order to investigate the performance of our incorporated L-SVC along with the benchmarked classifiers more accurately, we compute 3x3 confusion matrix due to multi-class dataset used as model input which has been illustrated in Table 5. P, Q and R are referred to the classes 1, 2 and 3 respectively. Using this matrix, the performance of each indicator (precision, recall, F1-score and accuracy) is calculated and later on, the results are compared. The confusion matrix is a useful tool to investigate function clustering techniques and to classify various classes of feature samples. In an ideal case, most of the feature samples are on the diagonal matrix and the rest of the matrix values are zero or near zero.

**Table 5:** Confusion matrix utilized in our study.

		Actual		
	Classes	P	Q	R
**Predicted**	**P**	TP- True P	FP1- False P and True Q	FP2- False P and True R
	**Q**	FQ1- False Q and True P	TQ- True Q	FQ2- False Q and True R
	**R**	FR1- False R and True P	FR2- False R and True Q	TR- True R

### Result analysis

**Fig 11:**
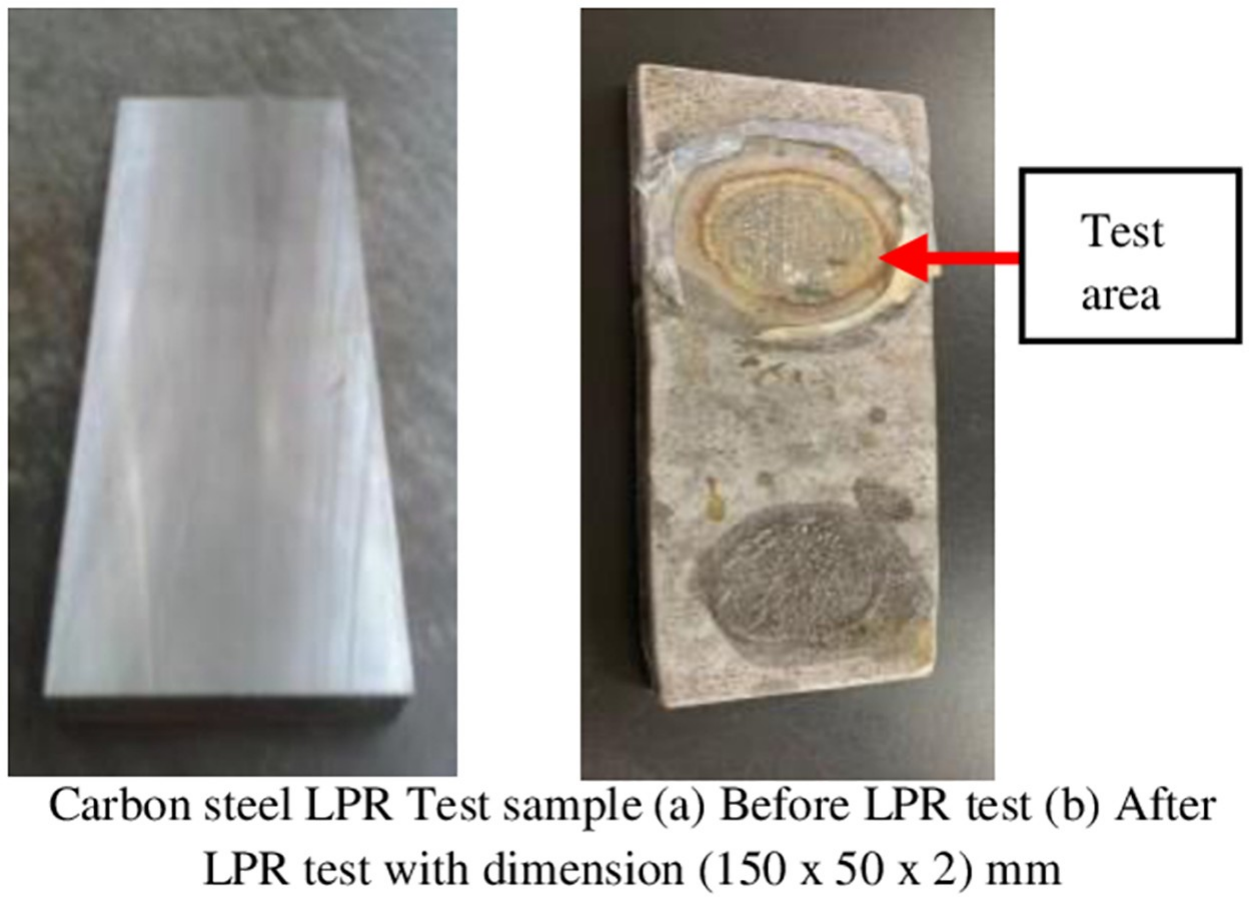
Specimen sample before and after LPR test.

Fig 11(a) shows the specimen before the LPR. The surface of the specimen was smooth as the surface finishing was done using sand blasting to remove any residue and defects. Later on, it is compared with the specimen test area after the LPR test as shown in Fig 11(b). The uniform corrosion was created during the LPR test where the metal surface was corroded in the form of circular shape following the hollow tube shape. The corroded surface area can be clearly seen despite any microscopic examination.

**Fig 12:**
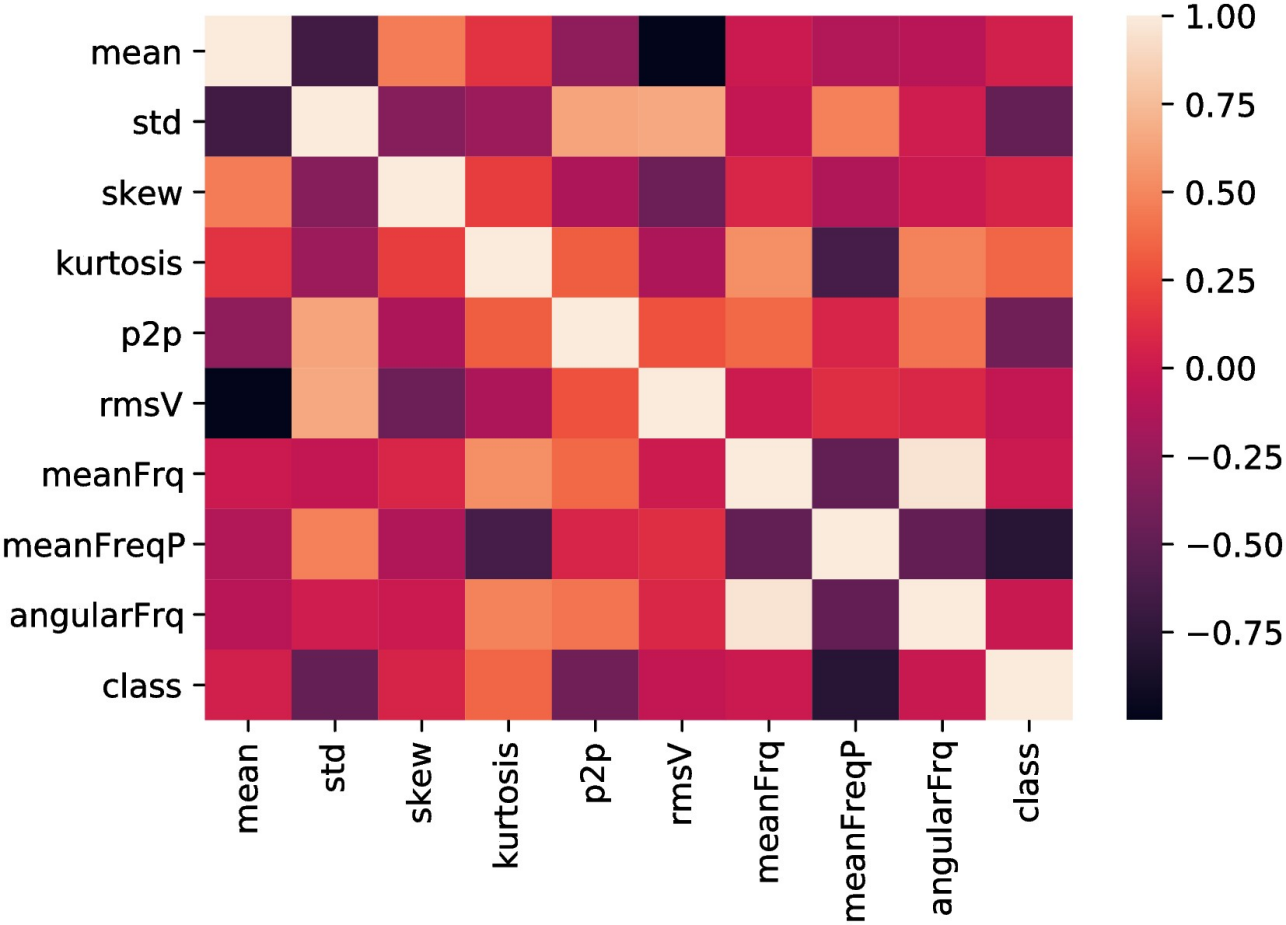
Feature heatmap of utilized AE dataset.

**Fig 13:**
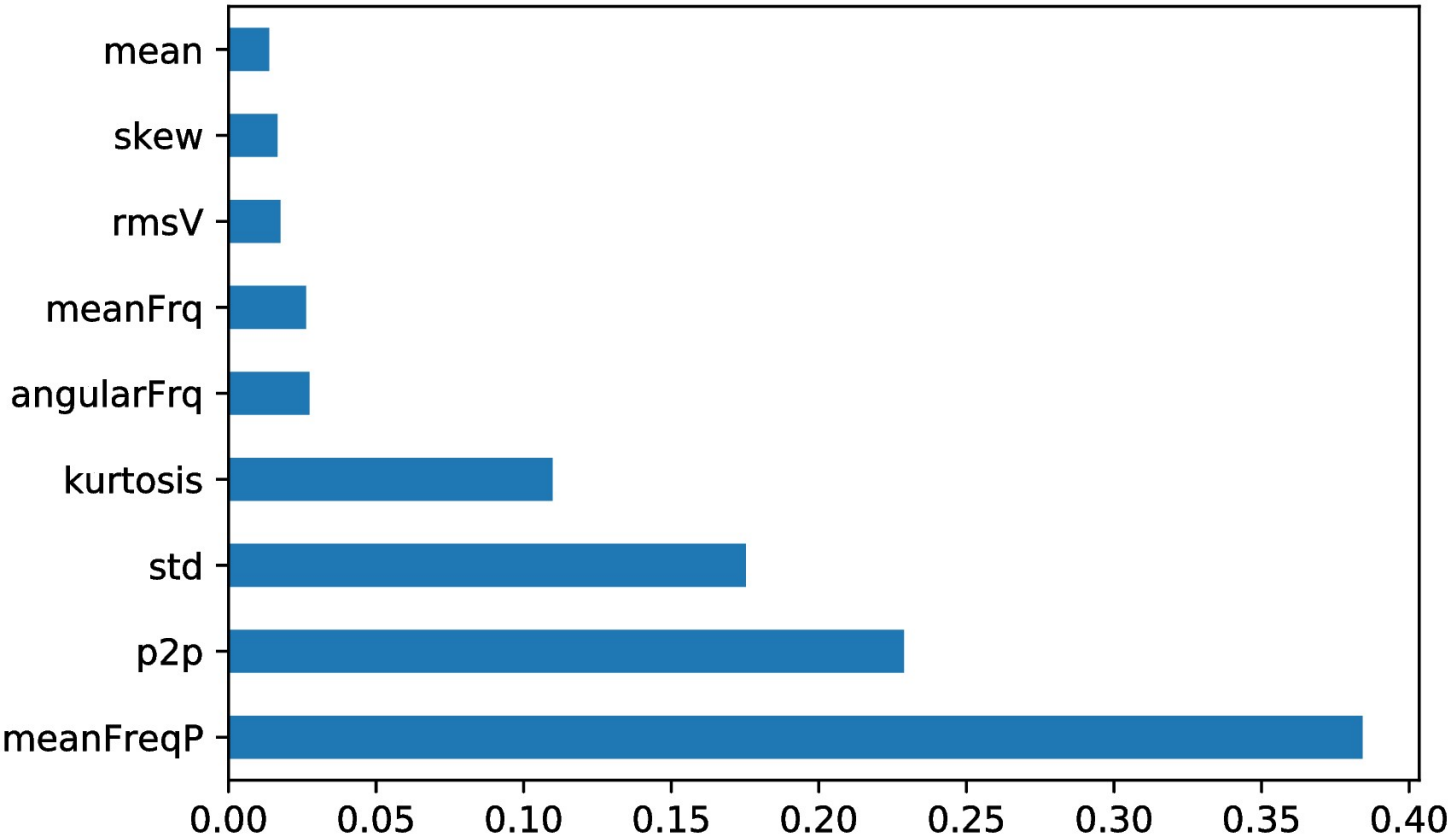
Feature importance of utilized AE dataset.

Fig 12 presents the features correlation matrix based on proposed feature extraction approach. The high positive correlation among features represents low feature importance and vice-versa. From this plot, it can be seen that the high correlation (close to 1.0) between statistical feature “mean” and frequency domain feature “angularFrq”. Thus, these features are less important for prediction of corrosion severity levels. However, most of the feature correlations are less than 0.25, which shows the effectiveness of appropriate feature selection for the prediction model. The importance of the selected features is shown in Fig 13 which is plotted based on the feature correlations among each. The vertical and horizontal lines present a number of features and correlation coefficient. The Analysis of Variance (ANOVA) of the extracted features has been carried out and presented in Table 6.

**Table 6:** ANOVA test results for the extracted features.

Source of Variation	SS	DF	MS	F-value	P-value	F-crit
**Within the groups**	5.3E+11	185	2.8E+9	1.28	0.008	1.19
**Between the groups**	3.7E+12	8	4.6E+11	205.88	3.9E-234	1.94
**Error**	3.3E+12	1480	2.2E+9			
**Total**	7.5E+12	1673				

**Fig 14:**
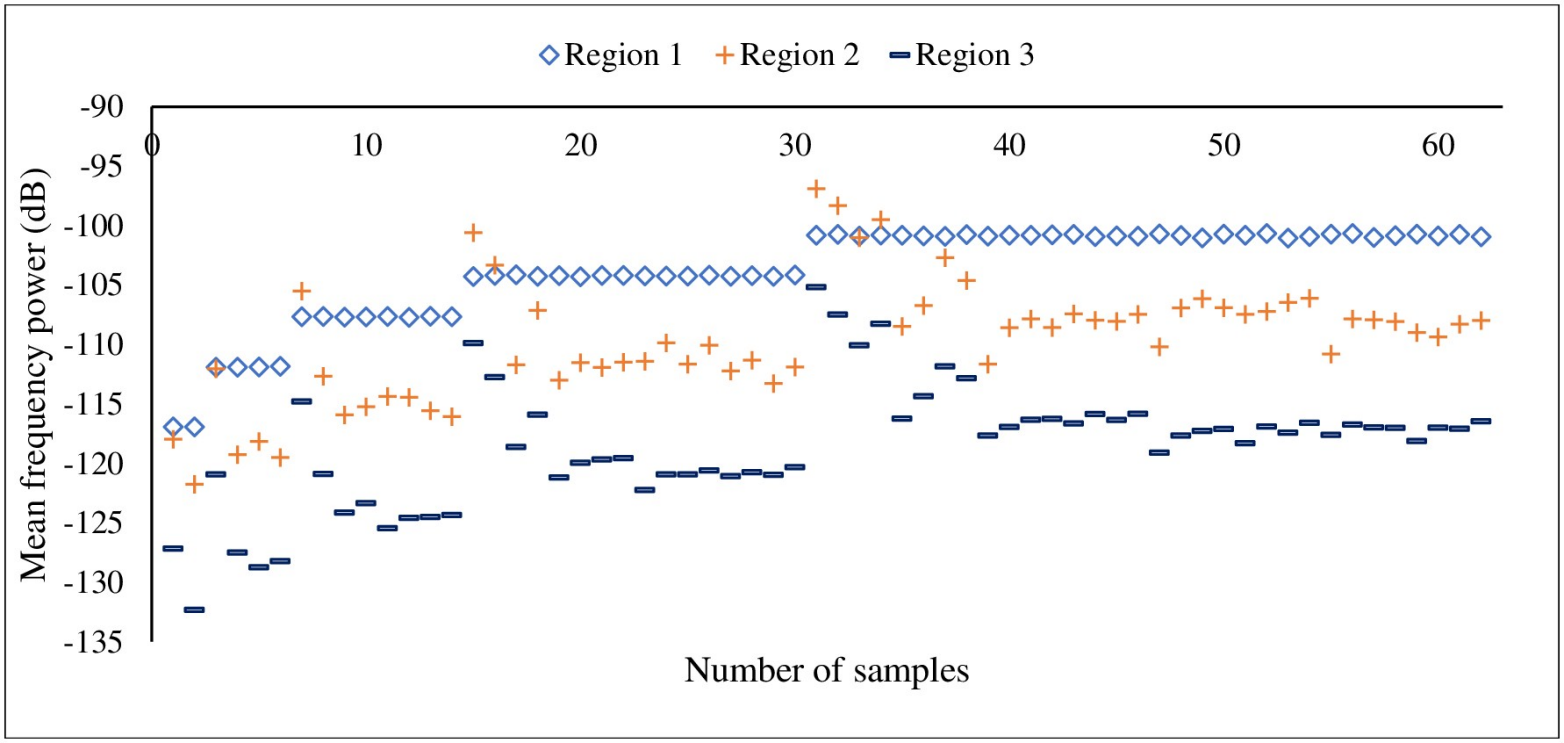
Mean frequency power, frequency-domain features for three stages of corrosion.

**Fig 15:**
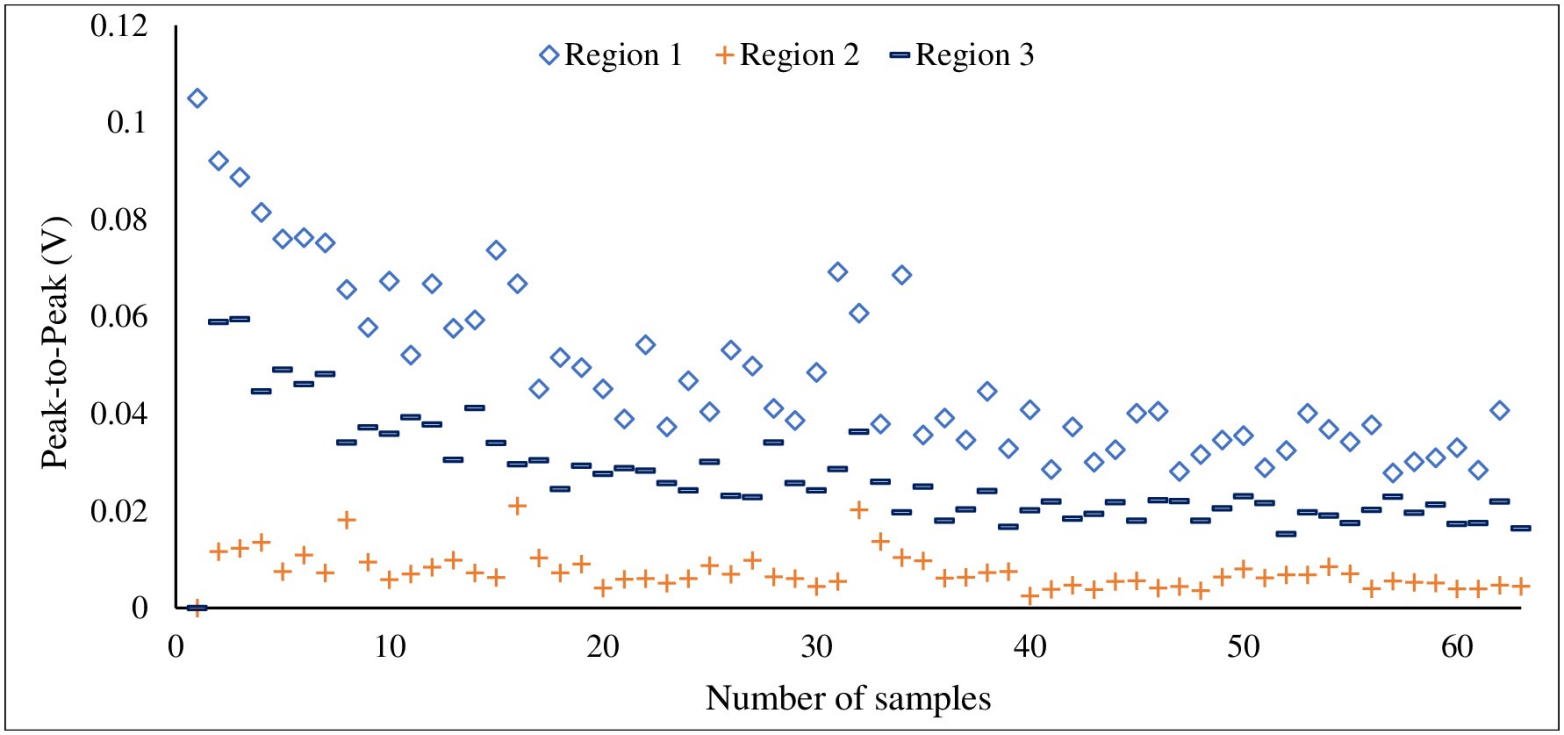
Peak-to-Peak (V), time-domain features for three stages of corrosion.

**Fig 16:**
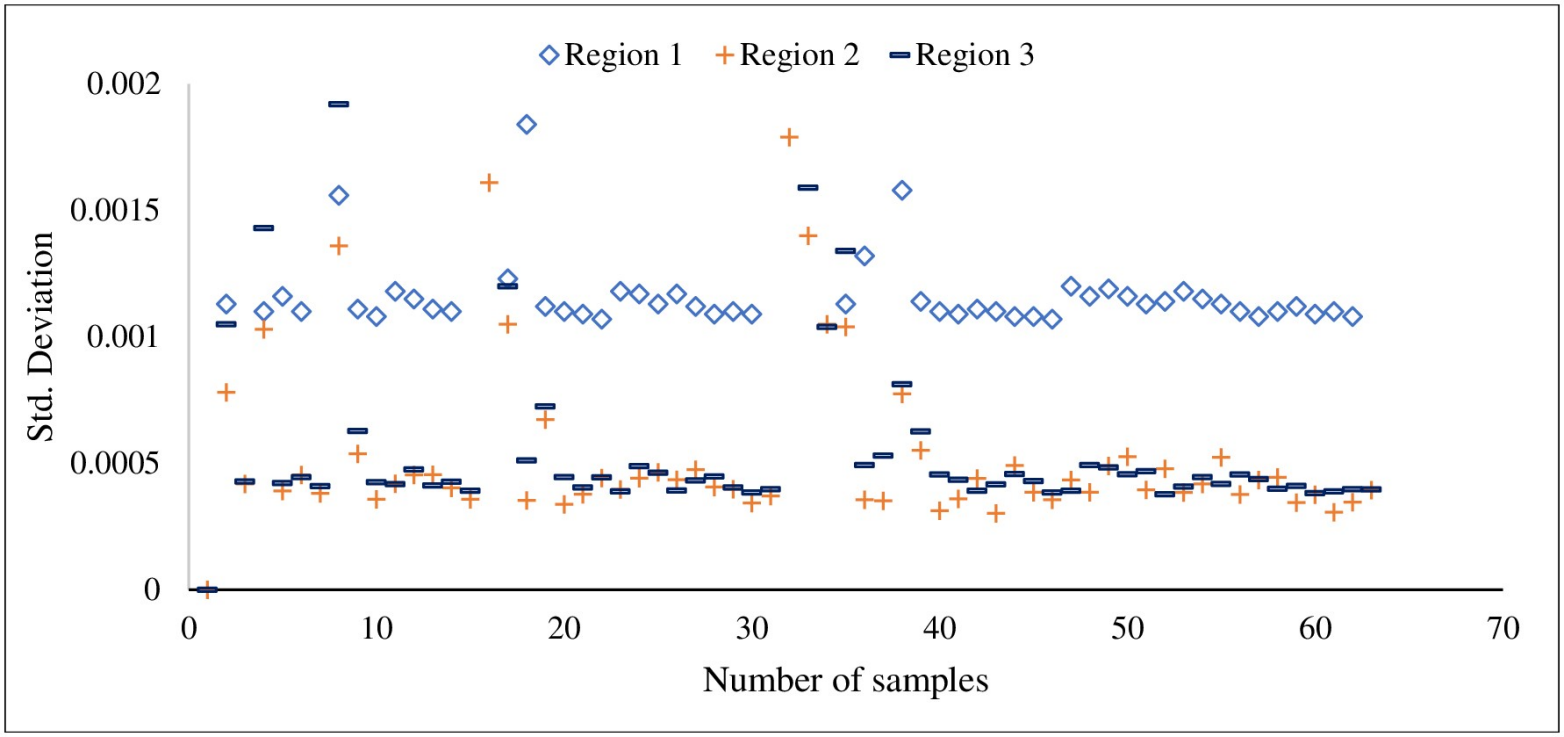
Standard deviation, statistical features for three stages of corrosion.

Here, the most important features of three different classes are plotted to analyse the feature discrimination among classes and the feature relationship on the corrosion process. Fig 14 presents the frequency-domain feature “mean frequency power”, which is the most important feature among others. The vertical line shows the mean frequency power for three different classes, and the horizontal line shows the number of feature samples for this particular feature. It can be seen from this plot that the feature samples are well discriminated among the classes and the feature samples of each class maintain a linear relationship with the corrosion process. A similar trend follows in Fig 15 where the second most important time-domain feature “peak-to-peak” for three classes of corrosion is visualized. Fig 16 shows the feature sample distribution of the third most important feature for three corrosion regions. Here, it can be observed that the less effectiveness in discriminating feature samples among classes due to high correlation among feature samples of different classes. However, there is still discrimination between “Region 1” and the other two regions, as well as a linear relationship between the samples of each region of corrosion. Moreover, the significant feature-extraction can be observed from the ANOVA test results where F-values decline the null hypothesis for both cases, within the groups and between the groups. Furthermore, the significant results are obtained for P-values that are less than the significance level (*p*-*value* < 0.05).

Based on our confusion matrix and above stated indicators’ formulas, the classification results of an individual class for the utilized L-SVC model as well as other SVM, DT, LR, and RF models are computed and stated in Table 7. The results are measured based on uniform corrosion dataset that were prepared from the LPR test experimental AE data. The better performance indicators listed in the table refers to the correctiveness of the prediction models. It can be seen from the table that L-SVC outperforms the benchmarked models with respect to the performance metrics. It achieves an average precision, recall, F1-score and AUC of 99.0%, 98.0%, 99.0% and 99.0% correspondingly. The main reason is that the L-SVC utilizes the kernel-trick to solve dual problems and sets the hyperplane at an optimal margin for class separation. Moreover, it generalizes the data well due to the linear behavior of the corrosion dataset. The DT model performs poorly for all indicators because this classifier is not well adapted to small variations in the data and is unable to generalize the data for prediction. It can be concluded that the L-SVC is more appropriate for the corrosion severity level prediction dataset due to its memory efficiency and low computations.

**Table 7:** Classification performance comparison between adopted L-SVC and benchmarked models.

Performance Metrics	Classes	DT	LR	RF	SVM	L-SVC
**Precision**	**Region 1**	0.64	1	0.85	0.93	1
	**Region 2**	0.96	0.93	1	0.89	0.96
	**Region 3**	0.46	1	0.78	1	1
**Avg**		0.69	0.98	0.88	0.93	0.99
**Recall**	**Region 1**	0.26	1	0.81	1	1
	**Region 2**	1	1	1	0.96	1
	**Region 3**	0.77	0.91	0.82	0.81 0.95	
**Avg**		0.68	0.97	0.88	0.93	0.98
**F1-score**	**Region 1**	0.37	1	0.83	0.96	1
	**Region 2**	0.98	0.96	1	0.92	0.98
	**Region 3**	0.58	0.95	0.8	0.90	0.98
**Avg**		0.64	0.97	0.88	0.93	0.99
**Actual**	**Region 1**	27	27	27	27	27
	**Region 2**	26	26	26	26	26
	**Region 3**	22	22	22	22	22
**Total**		75	75	75	75	75
**Predicted**	**Region 1**	11	27	26	29	27
	**Region 2**	27	28	26	28	27
	**Region 3**	37	20	23	18	21
**Total**		75	75	75	75	75
**AUC**	**Region 1**	1	1	0.93	1	1
	**Region 2**	0.99	0.99	1	0.99	0.99
	**Region 3**	1	1	0.92	1	1
**Avg**		0.99	0.99	0.95	0.99	0.99

Here, the performance of adopted L-SVC model as well as benchmarked classifiers are evaluated in terms of accuracy in training, testing, and cross-validation and associated errors. Table 8 presents the results obtained by L-SVC and compares them with the results computed by other benchmarked models. From the table, it can be seen that the L-SVC outperforms the other benchmarked models with respect to prediction accuracy and associated false prediction error. The L-SVC achieves the highest prediction accuracy of 99.0% along with the lowest false prediction error of 0.01 due to well separation with optimal margin and well adapted to the linear behavioral corrosion dataset. Thus, the L-SVC can be utilized for corrosion assessment, which offers better accuracy for multi-class corrosion severity level prediction than other evaluated models.

**Table 8:** Classification accuracy associated with error comparison between adopted L-SVC and benchmarked models.

Models	Training Accuracy	Testing Accuracy	10-Fold Cross Validation Accuracy	MAE
**DT**	0.98	0.67	-	0.65
**LR**	1	0.97	-	0.03
**RF**	1	0.88	-	0.24
**SVM**	1	0.93	-	0.08
**L-SVC**	1	0.99	0.98	0.01

**Fig 17:**
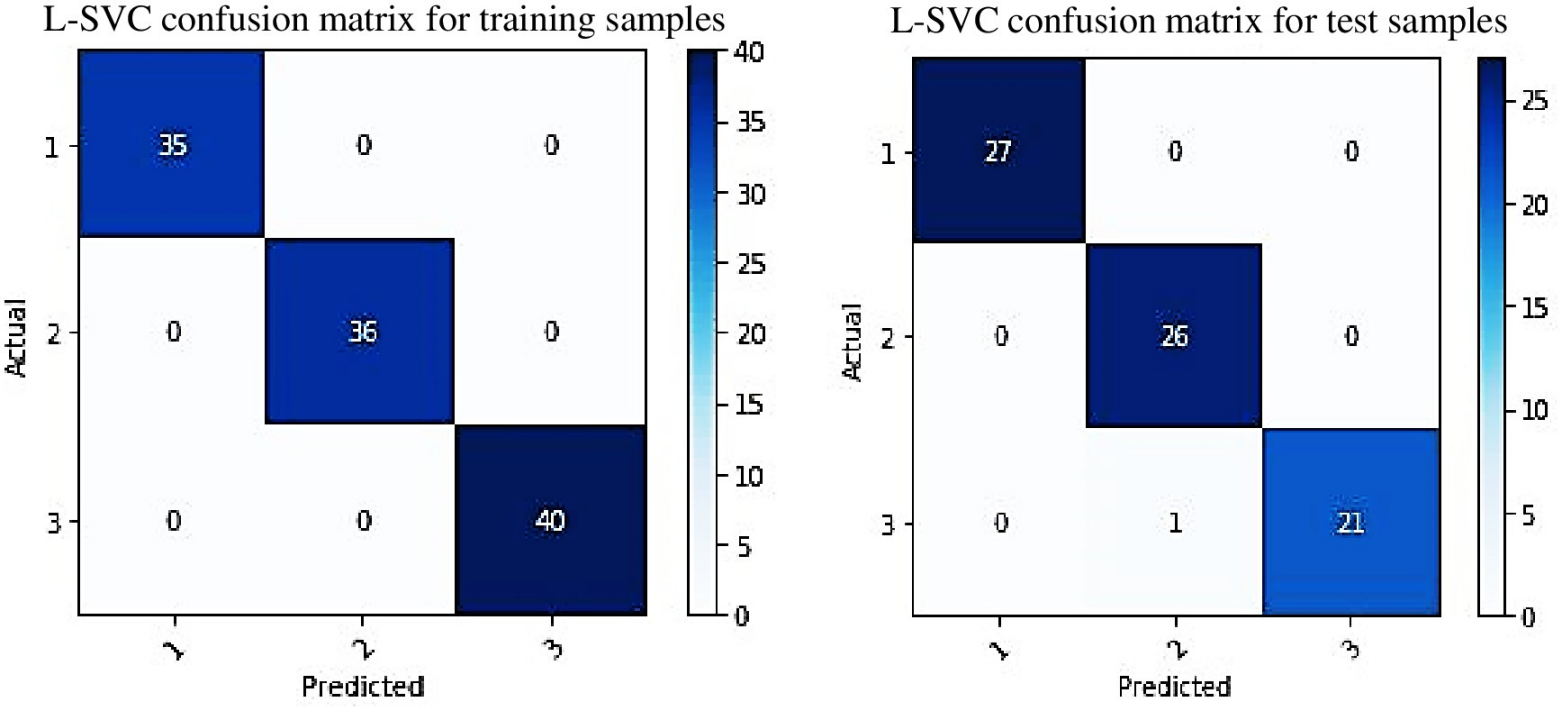
Confusion matrix of the adopted L-SVC.

In order to visualize the number of true predicted classes and false predicted classes utilizing the adopted classification model, the confusion matrices are computed for training and testing feature samples. Fig 17 shows two confusion matrices generated during training and testing samples based on the adopted L-SVC model. There are three actual and predicted classes (class: 1, 2 and 3) visualized in vertical and horizontal lines. The diagonal matrices represent the numbers of true predictions and the rest of the matrices present the false classification. There is no false classification number seen in the confusion matrix for all classes generated during training samples. However, even though there was a false prediction value in the confusion matrix during testing samples, the true prediction rates were 100% for class 1 and 2. It can be concluded that the L-SVC model is able to identify different levels of corrosion 100% during training and close to 100% during prediction, which confirms the effectiveness of the model in terms of corrosion severity level assessment.

### Summary of the findings

The proposed approach consists of three modules: First, the LPR test experiment for AE corrosion signal acquisition and categorization of uniform corrosion severity levels. Second, AE corrosion signal preprocessing and feature extraction for cleaning the various levels of corrosion AE signals and extracting several domains of AE features. Third, the classification model for predicting the severity level of corrosion based on our extracted feature dataset. The main findings of this work are summarized as follows:

- The LPR test experiment has been conducted to record and investigate the uniform corrosion AE data. Afterwords, the categorization of severity levels of corrosion has been performed based on the variation of corrosion rate over different time span and corrosion activity presented in Fig 9.
- Three different domains of multiresolution corrosion features have been extracted utilizing our new feature-extraction approach, WPT integrated with FFT, which is one of our main contributions to this article. The importance of extracted features is analysed based on the sum of coefficients for accurate prediction in Fig 13. An important feature can be considered when one class of feature samples is highly dissimilar to other classes of feature samples. Our feature-extraction approach can extract feature samples that vary from each other within the class and are dissimilar among the classes.
- The mathematical formulation of our adopted classifier L-SVC and the designation of L-SVC with the optimal parameters for our corrosion dataset have been carried out, which is our other main contribution. A 10-folds training dataset is used for cross-validation and the highest prediction accuracy of 99.0% is achieved compared to the benchmarked classifiers.
- The classification outcomes have been analysed based on various performance indicators. Moreover, our adopted model outperforms the other evaluated models in terms of most of the performance indicators which were observed.

## Concluding remarks

Our analysis and findings confirm the fact that the proposed machine learning approach is useful for uniform corrosion AE data acquisition, severity level assessment, multi-domains feature extraction and corrosion severity level prediction for early warning systems. LPR test experiments have been carried out on carbon-steel specimen to acquire different levels of uniform corrosion AE data that can be utilized to develop corrosion monitoring and severity estimation systems in various SHM applications. The WPT combined with the FFT decomposition method has been incorporated for multi-domains feature extraction with high variation among feature samples within the class. The high variation of feature groups can help classifiers predict appropriately with good accuracy. The L-SVC classification model has been adopted for our linear behavioral feature-sets which can predict the various severity levels of corrosion accurately. The ANOVA test results indicate the significance within and between the feature-groups where F-values (*F-value>1*) rejects the null hypothesis and P-values (*P-value<0.05*) are less than the significance level. The utilized L-SVC classifier achieves higher prediction accuracy of 99.0% than the accuracy of other benchmarked classifiers. The extension of this work will focus on various types of corrosion data acquisition and more variety of feature extraction to get a large dataset for our adopted L-SVC model performance evaluation.

## References

[1] Singh R. Corrosion control for offshore structures: cathodic protection and high-efficiency Coating. Gulf Professional Publishing; 2014.

[2] Wu K, Jung WS, Byeon JW. Acoustic emission of hydrogen bubbles on the counter electrode during pitting corrosion of 304 stainless steel. Materials Transactions. 2015;56(4):587–592. doi: 10.2320/matertrans.M2014373

[3] Patil S, Goyal S, Karkare B. Performance evaluation of accelerated corrosion techniques using electrochemical measurements and acoustic emission parameters. In: 2016 IEEE International Conference on Prognostics and Health Management (ICPHM). IEEE; 2016. p. 1–9.

[4] Prateepasen A. Pitting Corrosion Monitoring Using Acoustic Emission. Pitting corrosion. 2012; p. 43. doi: 10.5772/33127

[5] Droubi MG, Faisal NH. Application of acoustic emission to predict corrosion. 2017;.

[6] Saenkhum N, Prateepasen A, Keawtrakulpong P. Classification of Corrosion Detected by Acoustic Emission. In: ASME International Mechanical Engineering Congress and Exposition. vol. 37246; 2003. p. 33–41.

[7] De Masi G, Gentile M, Vichi R, Bruschi R, Gabetta G. Machine learning approach to corrosion assessment in subsea pipelines. In: OCEANS 2015-Genova. IEEE; 2015. p. 1–6.

[8] Liao K, Yao Q, Wu X, Jia W. A numerical corrosion rate prediction method for direct assessment of wet gas gathering pipelines internal corrosion. Energies. 2012;5(10):3892–3907. doi: 10.3390/en5103892

[9] Piotrkowski R, Castro E, Gallego A. Wavelet power, entropy and bispectrum applied to AE signals for damage identification and evaluation of corroded galvanized steel. Mechanical Systems and Signal Processing. 2009;23(2):432–445. doi: 10.1016/j.ymssp.2008.05.006

[10] Griffin JM, Chen X. Multiple classification of the acoustic emission signals extracted during burn and chatter anomalies using genetic programming. The International Journal of Advanced Manufacturing Technology. 2009;45(11):1152–1168. doi: 10.1007/s00170-009-2026-7

[11] Zhao J, Wang K, Guo Y. Acoustic emission signals classification based on support vector machine. In: 2010 2nd International Conference on Computer Engineering and Technology. vol. 6. IEEE; 2010. p. V6–300.

[12] Van Dijck G, Van Hulle M. Genetic algorithm for informative basis function selection from the wavelet packet decomposition with application to corrosion identification using acoustic emission. Chemometrics and Intelligent Laboratory Systems. 2011;107(2):318–332. doi: 10.1016/j.chemolab.2011.05.001

[13] Yu Y, Zhou L. Acoustic emission signal classification based on support vector machine. TELKOMNIKA Indonesian Journal of Electrical Engineering. 2012;10(5):1027–1032. doi: 10.11591/telkomnika.v10i5.1387

[14] Li J, Du G, Jiang C, Jin S. The classification of acoustic emission signals of 304 stainless steel during stress corrosion process based on K-means clustering. Anti-Corrosion Methods and Materials. 2012;. doi: 10.1108/00035591211210848

[15] Fahad M, Kamal K, Zafar T, Qayyum R, Tariq S, Khan K. Corrosion detection in industrial pipes using guided acoustics and radial basis function neural network. In: 2017 International Conference on Robotics and Automation Sciences (ICRAS). IEEE; 2017. p. 129–133.

[16] Sheikh MF, Kamal K, Rafique F, Sabir S, Zaheer H, Khan K. Corrosion detection and severity level prediction using acoustic emission and machine learning based approach. Ain Shams Engineering Journal. 2021;. doi: 10.1016/j.asej.2021.03.024

[17] Zheng Y, Zhou Y, Zhou Y, Pan T, Sun L, Liu D. Localized corrosion induced damage monitoring of large-scale RC piles using acoustic emission technique in the marine environment. Construction and Building Materials. 2020;243:118270. doi: 10.1016/j.conbuildmat.2020.118270

[18] Mangalgiri PD. Corrosion issues in structural health monitoring of aircraft. ISSS Journal of Micro and Smart Systems. 2019;8(1):49–78. doi: 10.1007/s41683-019-00035-z

[19] Beattie AG. Studies in the digital analysis of acoustic emission signals. Acoustical Society of America Journal. 1978;S1(64):S154. doi: 10.1121/1.2003922

[20] May MKANRMSM Zazilah, Nayan NA. Denoising of Hydrogen Evolution Acoustic Emission Signal Based on Non-Decimated Stationary Wavelet Transform. Processes. 2020;8(11):1460. doi: 10.3390/pr8111460

[21] Xu Y, Shao Y, Tian Y, Deng N. Linear Multi-class Classification Support Vector Machine. In: International Conference on Multiple Criteria Decision Making. Springer; 2009. p. 635–642.

